# Fat Oxidation Kinetics Is Related to Muscle Deoxygenation Kinetics During Exercise

**DOI:** 10.3389/fphys.2020.00571

**Published:** 2020-06-04

**Authors:** Anouck Zurbuchen, Stefano Lanzi, Ludovic Voirol, Cybele Barboza Trindade, Boris Gojanovic, Bengt Kayser, Nicolas Bourdillon, Xavier Chenevière, Davide Malatesta

**Affiliations:** ^1^Department of Neurosciences and Movement Science, Faculty of Science and Medicine, University of Fribourg, Fribourg, Switzerland; ^2^Institute of Sport Sciences of the University of Lausanne, Doctrine Selon Convention SSP-FBM, University of Lausanne, Lausanne, Switzerland; ^3^Division of Angiology, Heart and Vessel Department, Lausanne University Hospital, Lausanne, Switzerland; ^4^Sports Medicine Unit, Swiss Olympic Medical Center, Department for Locomotion, Lausanne University Hospital, Lausanne, Switzerland

**Keywords:** aerobic fitness, cycling, indirect calorimetry, Fat_max_, NIRS, breaking point

## Abstract

**Purpose:**

The present study aimed to determine whether whole-body fat oxidation and muscle deoxygenation kinetics parameters during exercise were related in individuals with different aerobic fitness levels.

**Methods:**

Eleven cyclists [peak oxygen uptake ($\dot{V}O{}_{2\,p\,e\,a\,k}$): 64.9 ± 3.9 mL⋅kg^–1^⋅min^–1^] and 11 active individuals ($\dot{V}O{}_{2\,p\,e\,a\,k}$: 49.1 ± 7.4 mL⋅kg^–1^⋅min^–1^) performed a maximal incremental cycling test to determine $\dot{V}O{}_{2\,p\,e\,a\,k}$ and a submaximal incremental cycling test to assess whole-body fat oxidation using indirect calorimetry and muscle deoxygenation kinetics of the vastus lateralis (VL) using near-infrared spectroscopy (NIRS). A sinusoidal (SIN) model was used to characterize fat oxidation kinetics and to determine the intensity (Fat_max_) eliciting maximal fat oxidation (MFO). The muscle deoxygenation response was fitted with a double linear model. The slope of the first parts of the kinetics (*a*_1_) and the breakpoint ([HHb]_BP_) were determined.

**Results:**

MFO (*p* = 0.01) and absolute fat oxidation rates between 20 and 65% $\dot{V}O{}_{2\,p\,e\,a\,k}$ were higher in cyclists than in active participants (*p* < 0.05), while Fat_max_ occurred at a higher absolute exercise intensity (*p* = 0.01). *a*_1_ was lower in cyclists (*p* = 0.02) and [HHb]_BP_ occurred at a higher absolute intensity (*p* < 0.001) than in active individuals. $\dot{V}O{}_{2\,p\,e\,a\,k}$ was strongly correlated with MFO, Fat_max_, and [HHb]_BP_ (*r* = 0.65–0.88, *p* ≤ 0.001). MFO and Fat_max_ were both correlated with [HHb]_BP_ (*r* = 0.66, *p* = 0.01 and *r* = 0.68, *p* < 0.001, respectively) and tended to be negatively correlated with *a*_1_ (*r* = -0.41, *p* = 0.06 for both).

**Conclusion:**

This study showed that whole-body fat oxidation and muscle deoxygenation kinetics were both related to aerobic fitness and that a relationship between the two kinetics exists. Individuals with greater aerobic fitness may have a delayed reliance on glycolytic metabolism at higher exercise intensities because of a longer maintained balance between O_2_ delivery and consumption supporting higher fat oxidation rates.

## Introduction

Regular endurance training leads to development of the muscle capillary network and marked changes in mitochondrial content and activity or in muscle fiber type composition (see Hawley et al., 2014 for review). Increased skeletal muscle capillarization enhances the substrate-exchange surface and fatty acid entry into myocytes (Sahlin et al., 2008). This leads to a greater fat oxidation capacity (Stisen et al., 2006; Sahlin et al., 2008) and delayed reliance on carbohydrates (Brooks and Mercier, 1994), resulting in a “glycogen-sparing effect” (Holloszy and Coyle, 1984) in endurance-trained athletes. The fat oxidation capacity may be a determining factor in endurance performance (Frandsen et al., 2017).

**FIGURE 1:**
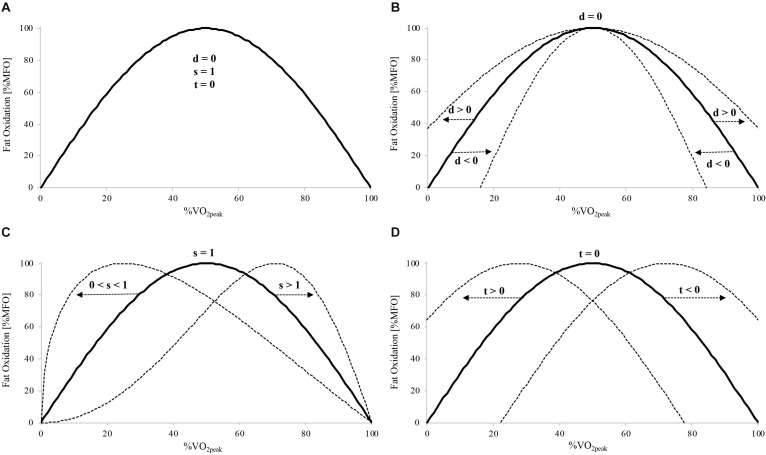
Schematic illustration of the three independent variables of the SIN model (dilatation [d], symmetry [s], and translation [t]) and their impact on the whole-body fat oxidation kinetics. Basic symmetric SIN curve with d = 0, s = 1, and t = 0 **(A)**. Changes in d **(B)**, s **(C)**, and t **(D)** and corresponding modifications in the basic symmetric SIN curve (dotted lines). MFO, maximal fat oxidation; $\dot{V}O{}_{2\,p\,e\,a\,k}$, peak oxygen uptake.

The whole-body fat oxidation rate measured during the incremental exercise test allows the estimation of an individual’s ability to oxidize fat (Achten et al., 2002; Cheneviere et al., 2009). Fat oxidation kinetics expressed as a function of exercise intensity gives a parabolic curve, with the maximal fat oxidation (MFO) rate occurring at an intensity defined as “Fat_max_” (Achten et al., 2002). Achten and Jeukendrup (2003) showed a greater fat oxidation rate over a wide range of intensities and a higher MFO in athletes with maximal oxygen uptake ($\dot{V}\,O_{2\,p\,e\,a\,k}$) above 65 mL⋅kg^–1^⋅min^–1^ compared to athletes with values below this level. This was concomitant with similar relative Fat_max_ values expressed as %$\dot{V}O{}_{2\,p\,e\,a\,k}$ in both groups. Similar results were found when comparing trained with untrained men (Lima-Silva et al., 2010). In addition to MFO and Fat_max_, Cheneviere et al. (2009) developed a sinusoidal (SIN) model with three independent variables (*dilatation*, *symmetry*, and *translation*) to mathematically characterize the shape of the whole-body fat oxidation kinetics as a function of exercise intensity (see Figure 1 for a graphic representation of these three variables). As aerobic fitness influences fat oxidation capacity, the greater MFO and fat oxidation rates across exercise intensities found in trained athletes (Achten and Jeukendrup, 2003; Stisen et al., 2006; Lima-Silva et al., 2010) may result in an upward and more dilated (greater *dilatation*) projection of the fat oxidation kinetics compared to less-trained individuals.

A better oxygen (O_2_) delivery and utilization into mitochondria may partly explain the greater fat oxidation capacity in endurance-trained athletes. In fact, endurance training leads to improved muscle blood perfusion. The increased muscular blood flow (${\dot{Q}}_{\text{m}}$) provides more O_2_ to the working muscles, allowing a better balance between O_2_ delivery and muscular O_2_ utilization ($\dot{V}O{}_{2\,m}$) (Koga et al., 2014). Given the exclusive reliance on O_2_ for fat use as an energy substrate, it would be expected that differences in fat oxidation capacity would be mirrored by differences in the regulation of muscle O_2_ supply. The ${\dot{Q}}_{\text{m}}$ / $\dot{V}O{}_{2\,m}$ ratio is considered an important determinant of aerobic performance and seems to be linked to the aerobic capacity (Ferreira et al., 2007; Boone et al., 2012). This ratio can be estimated by integrating the near-infrared spectroscopy (NIRS), a non-invasive technique used to assess tissue oxygenation levels and changes during exercise (see Grassi and Quaresima, 2016 for review), with the pulmonary $\dot{V}O{}_{2}$ data. NIRS measures variations in the deoxygenated hemo- and myoglobin concentration (Δ[HHb]). Δ[HHb] is considered to be an indicator of the balance between O_2_ delivery and consumption, providing a solid estimation of the dynamic profile of O_2_ extraction within the area of NIRS interrogation (DeLorey et al., 2003). Some authors acknowledge that the dynamic relationship between ${\dot{Q}}_{\text{m}}$ and $\dot{V}O{}_{2\,m}$ follows a sigmoid model during incremental exercise: ${\dot{Q}}_{\text{m}}$ kinetics is initially faster than $\dot{V}O{}_{2\,m}$ kinetics and slows progressively to approximate $\dot{V}O{}_{2\,m}$ kinetics as the exercise intensity increases (Ferreira et al., 2007; Boone et al., 2009). However, it seems that the Δ[HHb] response may be better described by a double linear model (Spencer et al., 2012) because it would more precisely explain the physiological mechanisms underlying the Δ[HHb] kinetics (see Boone et al., 2016b for review). According to this model, Δ[HHb] increases linearly with work load until a breakpoint ([HHb]_BP_), followed by a near-plateau. Although somewhat controversial (Broxterman et al., 2018; Keir et al., 2018), [HHb]_BP_ potentially reflects a physiological response, such as maximal lactate steady state, critical power or the respiratory compensation point (RCP). Since endurance athletes have good oxidative capacity, their [HHb]_BP_ may appear at higher absolute (McNarry et al., 2015) and relative intensities (Boone et al., 2009, 2016a; Murias et al., 2013), reflecting delayed reliance on anaerobic ATP production. Therefore, the first part of the muscular deoxygenation kinetics may present a rightward shift and a slower slope in endurance athletes who characteristically have a higher aerobic capacity compared to sedentary individuals (Boone et al., 2016a).

The adaptations to endurance training affecting aerobic fitness seem to influence both muscular deoxygenation and fat oxidation kinetics. However, to the best of our knowledge, the relationship between the two kinetics has never been investigated in healthy individuals of various aerobic fitness levels. Therefore, the purpose of the present study was to determine whether whole-body fat oxidation and muscle deoxygenation kinetics parameters during exercise were related in individuals with different aerobic fitness levels (trained cyclists versus active individuals). It was hypothesized that (i) cyclists would have shallower muscular deoxygenation kinetics with a right-shifted [HHb]_BP_, as well as upward and more dilated (greater *dilatation*) fat oxidation kinetics and (ii) a direct relationship between both kinetics would exist, with shallower muscular deoxygenation kinetics corresponding to higher fat oxidation rates.

## Materials and Methods

### Participants

Twenty-two healthy male subjects [11 daily trained cyclists and triathletes ($\dot{V}O{}_{2\,p\,e\,a\,k}$ ≥ 60 mL⋅kg^–1^⋅min^–1^) and 11 non-specifically trained but active individuals] aged between 20 and 39 years participated in this study (Table 1). They declared that they were non-smokers, disease-free, and not taking any medication. All subjects were fully informed about the protocol and its possible risks and benefits, and they all provided written consent. The protocol was conducted according to the Declaration of Helsinki and was approved by the local ethics committee (Cantonal Swiss Ethics Committees on research involving humans).

**TABLE 1:** Participant physical characteristics and aerobic fitness parameters.

	Cyclists (*n* = 11)	Active group (*n* = 11)
**Participant characteristics**		
Age [years]	27.4 ± 5.1	28.7 ± 5.1
Height [m]	1.83 ± 0.03	1.80 ± 0.07
Weight [kg]	72.3 ± 2.8	77.7 ± 8.1*
BMI [kg⋅m^–2^]	21.7 ± 0.9	23.9 ± 2.4*
FFM [kg]	64.2 ± 2.7	65.7 ± 5.1
FM [kg]	8.1 ± 2.9	12.0 ± 4.0*
FM [%]	11.1 ± 3.7	15.2 ± 3.8*
**Maximal exercise test**		
MAP [W⋅kg^–1^]	5.5 ± 0.4	4.1 ± 0.6*
$\dot{V}O{}_{2\,p\,e\,a\,k}$ [mL⋅kg^–1^⋅min^–1^]	64.9 ± 3.9	49.1 ± 7.4*
RCP [mL⋅kg^–1^⋅min^–1^]	56.1 ± 4.2	41.7 ± 7.4*
RCP [%$\dot{V}O{}_{2\,p\,e\,a\,k}$]	86.4 ± 2.1	84.8 ± 5.4
VT [mL⋅kg^–1^⋅min^–1^]	43.1 ± 4.5	30.4 ± 7.0*
VT [%$\dot{V}O{}_{2\,p\,e\,a\,k}$]	66.4 ± 4.7	61.5 ± 7.7

Data are presented as the mean ± SD. BMI, body mass index; FM, fat mass; FFM, fat-free mass; MAP, maximal aerobic power; $\dot{V}O{}_{2\,p\,e\,a\,k}$, peak oxygen uptake; VT, ventilatory threshold; RCP, respiratory compensation point. *Significant difference between groups (*p* ≤ 0.05).

### Experimental Design

Each subject completed three test sessions. In the first session, anthropometric measurements (i.e., height, body mass, and composition) were taken. Each subject then performed a maximal incremental exercise test on a cycle ergometer. For the remaining two sessions, the subjects performed two identical submaximal incremental exercise tests [i.e., until a respiratory exchange ratio (RER) of 1.0]. Sessions 2 and 3 were identical and separated by a minimum of 7 days and a maximum of 10 days. The pre-experimental conditions were controlled to standardize measurements and to minimize inter- and intra-individual variations. Both tests were performed in the morning after a minimum 10-h overnight fast, after 3 days of a balanced diet (2300–2500 kcal/day), with no caffeine and no alcohol during the last 48 h, and the volunteers were asked to refrain from vigorous exercise during the day before the experimental trials. Before the beginning of the testing session, all the subjects confirmed that they had followed the nutritional indications, and the food diary was checked by the experimenters.

### Assessments

#### Anthropometric Measurements

Height, body mass, and composition were assessed. Body composition [i.e., fat mass (FM) and lean mass] was estimated from the Durnin and Womersley’s skinfold-thickness measurement method at four sites (Durnin and Womersley, 1973).

#### Aerobic Fitness

To determine peak oxygen uptake ($\dot{V}O{}_{2\,p\,e\,a\,k}$), maximal aerobic power (MAP), ventilatory threshold (VT), and RCP, each subject performed an incremental exercise test to exhaustion on a cycle ergometer (Ebike Basic BPlus, General Electric, Niskayuna, NY, United States). After a 3-min rest on the ergometer, the test began with a 5-min warm-up at 60 W. The power output was then increased by 30 W every minute until exhaustion (Malatesta et al., 2009). Pedaling frequency was maintained between 70 and 90 revolutions per minute (rpm). The maximal incremental test ended when participants failed to maintain a pedaling frequency > 60 rpm despite maximum effort and verbal encouragement. Gas exchanges were continuously measured with a breath-by-breath online system (OxyconPro, Jaeger, Germany). The device was calibrated in three steps before each test: (1) ambient air analysis; (2) gas analyzer calibration with a known gas mixture (16.00% O_2_, 5.02% CO_2_); and (3) volume turbine calibration with a 3-L syringe. $\dot{V}O{}_{2}$$\dot{V}O{}_{2\,p\,e\,a\,k}$ corresponded to the highest $\dot{V}O{}_{2}$ value computed from a 15-s rolling average during the test. The power output corresponding to $\dot{V}O{}_{2\,p\,e\,a\,k}$ or to the lowest power output corresponding to the plateau of $\dot{V}O{}_{2}$ (i.e., a plateau of $\dot{V}O{}_{2}$ corresponded to an increase of no more than 2 mL⋅kg^–1^⋅min^–1^ with an increase of workload during the latter stages of the test) was considered as the MAP. The VT and RCP were estimated by three blinded and independent investigators: ventilatory equivalent method was used to determine both thresholds (Wasserman et al., 1973), while V-slope method was used to confirm VT (Beaver et al., 1986).

#### Whole-Body Fat Oxidation Kinetics

Both submaximal experimental trials served to determine whole-body fat oxidation kinetics. Resting values were measured during a 10-min seated-rest. This was followed by a 10-min warm-up at 20% MAP. The workload was then increased by 7.5% MAP for the active group and 10% MAP for cyclists every 5 min until the end of the stage during which RER was 1.0 (Cheneviere et al., 2011). For the submaximal exercise test, pedaling frequency was maintained between 70 and 90 rpm. Average values for $\dot{V}O{}_{2}$ and $\dot{V}\,C\,O_{2}$ were calculated during the last minute of warm-up and during the last minute of each stage of the test. The values obtained during each test session were then ensemble-averaged to reduce the intra-individual variability of fat oxidation rates using indirect calorimetry (Croci et al., 2014). Fat oxidation rates were calculated using stoichiometric equations (Frayn, 1983). The results of the submaximal incremental tests were used to calculate the fat oxidation rates over a wide range of exercise intensities. To model whole-body fat oxidation kinetics, represented as a function of exercise intensity, and to determine Fat_max_ and MFO, the SIN model was used (Cheneviere et al., 2009). This model includes three independent variables, representing the main quantitative characteristics of the curve, namely, *dilatation*, *symmetry*, and *translation*, indicated as *d*, *s*, and *t*, respectively, as well as pi (π) corresponding to *∼*3.14 and a constant of intensity named *K*, which corresponds to (π/100):

(1) %MFO=sin[{π1/sπ+2⁢d(K⋅%V.O+2⁢p⁢e⁢a⁢kd+t)}s]

where *dilatation* refers to the degree of dilation or retraction of the curve, *symmetry* modifies the symmetry of the standard basic sine curve, and *translation* refers to the right-left shift of the whole curve toward the abscissa axis (Figure 1). To fit the fat oxidation rates and to model the fat oxidation kinetics, the three variables were independently changed using an iterative procedure by minimizing the sum of the mean squares (SMS) of the differences between the estimated energy derived from lipid (E_lipid_) based on the SIN model and the calculated values of E_lipid_, as described in a previous study (Cheneviere et al., 2009). For each subject, Fat_max_ was calculated by differentiation of the SIN model equation, and the fat oxidation rate was determined at every 5% $\dot{V}O{}_{2\,p\,e\,a\,k}$ between 20 and 85% $\dot{V}O{}_{2\,p\,e\,a\,k}$.

#### Muscle Deoxygenation Kinetics

During both submaximal exercise tests, deoxyhemoglobin (HHb), oxyhemoglobin (O_2_Hb), and total hemoglobin (Hb_tot_) concentrations were continuously measured in the right *vastus lateralis* (VL) muscle using NIRS (Oxymon Mk III, Artinis Medical System, Netherlands) at 50 Hz to determine muscle deoxygenation. The NIRS probe contains a three-diode emitter operating at wavelengths of 764 and 860 nm and a detector positioned 40 mm away from the emitter. After calibration of the device, the probe was positioned on shaved and cleaned skin over the right VL muscle, halfway between the great trochanter and lateral femoral condyle, parallel to the major axis of the thigh. The VL muscle was chosen to assess exercise-induced changes in [HHb] concentrations since this muscle is highly active during cycling (Laplaud et al., 2006). After this placement, pen marks were made on the skin at the margins of the probe to verify that it did not move during the trial and to enable the exact same placement in the next test session. The probe was then firmly fixed to the thigh with tape, a bandage, and the aid of the subject’s shorts, reducing the influence of ambient light on the measurements. Light absorption changes are converted into [HHb] concentration changes using a modified Lambert–Beer law in which a path-length factor is incorporated to correct for the scattering of photons in the tissue (Ferrari et al., 1997). A differential path-length factor of 6.5 was used (Duncan et al., 1995). Changes in [HHb] concentrations were downsampled to 1 Hz and then time aligned to the onset of exercise and ensemble-averaged. Average values for [HHb] were calculated during the last minute of warm-up and during the last minute of every stage of the incremental submaximal test. The values obtained were then normalized so that 0% represented the steady-state [HHb] value during warm-up and 100% represented the highest average [HHb] value observed in the exercise test (i.e., %Δ[HHb]). The normalized values were plotted as a function of absolute ($\dot{V}O{}_{2}$) and relative exercise intensity (%$\dot{V}O{}_{2\,p\,e\,a\,k}$) and were analyzed by means of a double linear model (Spencer et al., 2012; Boone et al., 2016a) as follows:

%⁢Δ⁢[HHb]={a1⋅x+b           ifx≤[HHb]  BP(2)a1⋅x+a2(x-BP)+b  ifx>[HHb]  BP(3)

where *a*_1_ is the slope of the linear regression before the breaking point ([HHb]_BP_); *b* is the *y*-intercept of the linear regression before the [HHb]_BP_; and *a*_2_ is the change in the slope of the linear regression after the [HHb]_BP_. The model parameters were estimated using an iterative procedure by minimizing the SMS of the differences between the estimated %Δ[HHb] based on the double linear model and the measured values of %Δ[HHb]. The slope of the linear regression after the [HHb]_BP_ (*a*_3_) was then calculated as the sum of *a*_1_ and *a*_2_.

### Statistical Analysis

Data are expressed as the mean ± SD for all variables. The sample size of 11 participants per group was calculated based on previously published data (Nordby et al., 2006). Before each statistical test, the normality and equality of variances of the data were verified using the Shapiro–Wilk test and Levene test, respectively. Differences in physical characteristics, aerobic fitness, SIN model, and double linear parameters between trained cyclists and active individuals were assessed using a two-samples *t*-test or the Mann–Whitney test for non-parametric data. Independent correlations between the parameters of whole-body fat oxidation kinetics and those of the double linear model of %Δ[HHb] were performed by using Pearson (*r*) or Spearman (*rho*) correlation coefficients. Furthermore, a two-way repeated-measures mixed design ANOVA (group × intensity) was used to compare the change in fat oxidation rates with exercise intensity (in a range from 20 to 85% of $\dot{V}O{}_{2\,p\,e\,a\,k}$) between both groups. The level of significance was set at *p* ≤ 0.05. Data analyses were all performed using RStudio (version 0.98.1103).

## Results

### Participants

The participants’ physical characteristics and their aerobic fitness parameters are listed in Table 1. The age and height were similar in both groups (*p* = 0.40 and *p* = 0.36, respectively). In comparison with the active group, cyclists had a lower body mass index (BMI) (*p* = 0.02) and FM (in % and in kg) (*p* = 0.02 for both), while their $\dot{V}O{}_{2\,p\,e\,a\,k}$ and MAP were higher (*p* < 0.001), confirming that the aerobic fitness level was different between the two experimental groups. Although no difference was found in the RCP and VT expressed as % $\dot{V}O{}_{2\,p\,e\,a\,k}$ between the two groups (*p* = 0.40 and *p* = 0.09, respectively), the absolute values of the VT and RCP were higher in cyclists (*p* < 0.001).

### Whole-Body Fat Oxidation Kinetics

**FIGURE 2:**
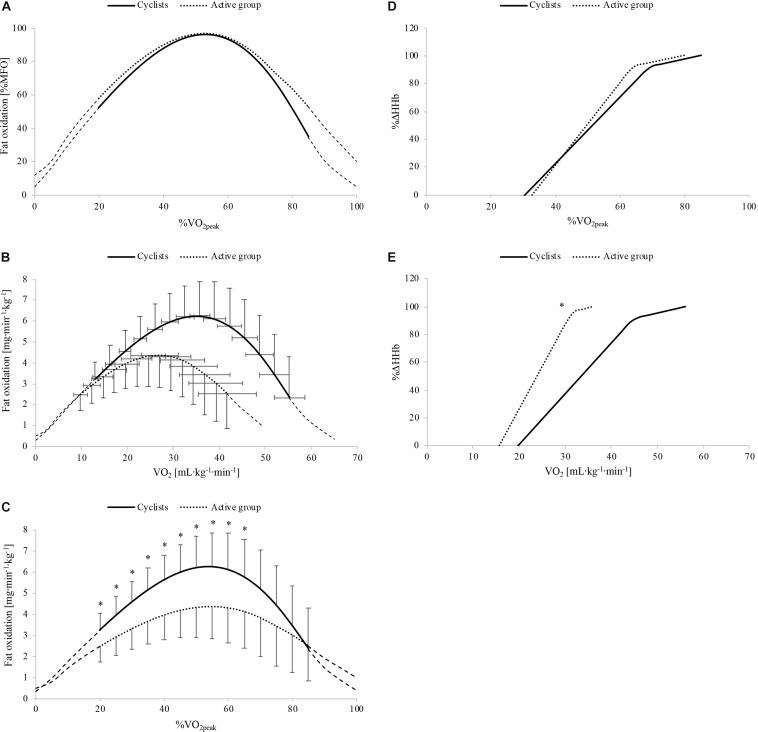
Whole-body fat oxidation kinetics in relative **(A)** and absolute **(B,C)** values and muscle deoxygenation kinetics as a function of relative **(D)** and absolute **(E)** exercise intensity in cyclists (black line) and active people (black dots). Δ[HHb], variation in deoxygenated hemo- and myoglobin concentration; MFO, maximal fat oxidation; $\dot{V}O{}_{2\,p\,e\,a\,k}$, peak oxygen uptake. ^∗^Significant difference between the cyclist and active group (*p* ≤ 0.05).

Whole-body fat oxidation kinetics, expressed in relative and absolute values, are shown in Figures 2A–C, respectively. The absolute fat oxidation rate (mg⋅kg^–1^⋅min^–1^) was higher in the cyclist group (*p* = 0.03) than that in the active group for the exercise intensities between 20 and 65% $\dot{V}O{}_{2\,p\,e\,a\,k}$ (*p* ≤ 0.046; Figure 2C). Table 2 shows the whole-body fat oxidation parameters and SIN model variables. MFO and Fat_max_ in mL⋅kg^–1^⋅min^–1^ were higher in cyclists than in active individuals (*p* = 0.01 for both). However, no difference was found when Fat_max_ was expressed as %$\dot{V}O{}_{2\,p\,e\,a\,k}$ (*p* = 0.77). The *dilatation* was greater in the active group than in the cyclist group (*p* = 0.003), while no difference in *symmetry* or *translation* was found between the two groups (*p* = 0.92 and *p* = 0.64, respectively).

**TABLE 2:** Whole-body fat oxidation parameters and SIN model variables.

	Cyclists (*n* = 11)	Active group (*n* = 11)
MFO [mg⋅kg^–1^⋅min^–1^]	6.5 ± 1.5	4.5 ± 1.6*
Fat_max_ [mL⋅kg^–1^⋅min^–1^]	33.9 ± 6.6	26.2 ± 5.8*
Fat_max_ [%$\dot{V}O{}_{2\,p\,e\,a\,k}$]	52.1 ± 9.3	53.2 ± 8.3
*Dilatation*	0.03 ± 0.16	0.25 ± 0.15*
*Symmetry*	1.36 ± 0.49	1.34 ± 0.53
*Translation*	0.18 ± 0.22	0.11 ± 0.41

Data are presented as the mean ± SD. MFO, maximal fat oxidation; Fat_max_, intensity at which MFO occurs; $\dot{V}O{}_{2\,p\,e\,a\,k}$, peak oxygen uptake. *Significant difference between groups (*p* ≤ 0.05).

Maximal fat oxidation was positively correlated with $\dot{V}O{}_{2\,p\,e\,a\,k}$ (*r* = 0.65; *p* = 0.001), MAP (*rho* = 0.43; *p* = 0.045), and VT in mL⋅kg^–1^⋅min^–1^ (*r* = 0.74; *p* < 0.001) and in %$\dot{V}O{}_{2\,p\,e\,a\,k}$ (*r* = 0.68; *p* = 0.001) and was correlated with RCP only when expressed in mL⋅kg^–1^⋅min^–1^ (*r* = 0.62; *p* = 0.002). Similarly, Fat_max_ (mL⋅kg^–1^⋅min^–1^) was also positively correlated with $\dot{V}O{}_{2\,p\,e\,a\,k}$ (*r* = 0.73; *p* < 0.001) and VT in mL⋅kg^–1^⋅min^–1^ (*r* = 0.77; *p* < 0.001) and in %$\dot{V}O{}_{2\,p\,e\,a\,k}$ (*r* = 0.59; *p* = 0.004) and was correlated with RCP only when expressed in mL⋅kg^–1^⋅min^–1^ (*r* = 0.69; *p* < 0.001). In contrast, the relative value of Fat_max_ was not correlated with any aerobic fitness parameters.

### Muscle Deoxygenation Kinetics

The %Δ[HHb] response as a function of relative and absolute exercise intensity is shown in Figures 2D,E, respectively. The parameters of the double linear model as a function of relative exercise intensity for the two experimental groups are presented in Table 3. These values indicate that the curve of muscle deoxygenation kinetics had a shallower slope with a right-shifted [HHb]_BP_ in cyclists compared to that in active individuals. In fact, *a*_1_ was lower in the cyclist group than in the active group (*p* = 0.02). The [HHb]_BP_ occurred at a higher absolute intensity (in mL⋅kg^–1^⋅min^–1^) but not at a higher relative intensity (in %$\dot{V}O{}_{2\,p\,e\,a\,k}$) in cyclists than in active individuals (*p* < 0.001 and *p* = 0.098, respectively). The [HHb]_BP_ expressed in mL⋅kg^–1^⋅min^–1^ was strongly and positively correlated with $\dot{V}O{}_{2\,p\,e\,a\,k}$ (*r* = 0.88; *p* < 0.001), MAP (*rho* = 0.80; *p* < 0.001), VT (in mL⋅kg^–1^⋅min^–1^ and in %$\dot{V}O{}_{2\,p\,e\,a\,k}$: *r* = 0.83; *p* < 0.001 and *r* = 0.50; *p* = 0.02, respectively), and RCP (in mL⋅kg^–1^⋅min^–1^, *r* = 0.87; *p* < 0.001).

**TABLE 3:** Muscle deoxygenation kinetics parameters.

	Cyclists (*n* = 11)	Active group (*n* = 11)
*a*_1_	2.39 ± 0.39	2.96 ± 0.66*
*a*_2_	−1.88 ± 0.69	−2.47 ± 0.92
*a*_3_	0.51 ± 0.44	0.49 ± 0.44
*B*	−72.59 ± 14.93	−96.81 ± 23.94*
[HHb]_BP_ [mL⋅kg^–1^⋅min^–1^]	44.8 ± 3.1	31.4 ± 4.6*
[HHb]_BP_ [%$\dot{V}O{}_{2\,p\,e\,a\,k}$]	69.0 ± 5.6	64.0 ± 7.7

Data are presented as the mean ± SD. *a*_1_ and *a*_3_, slopes of the first and second parts of the curve of the double linear model, respectively; *a*_2_, change in the slope between the first and the second parts of the curve of the double linear model; *b*, *y*-intercept of the first part of the curve of the double linear model; [HHb]_BP_, breakpoint of the curve of the double linear model; $\dot{V}O{}_{2\,p\,e\,a\,k}$, peak oxygen uptake. *Significant difference between groups (*p* ≤ 0.05).

### Correlations Between Fat Oxidation and Muscle Deoxygenation Kinetics

**FIGURE 3:**
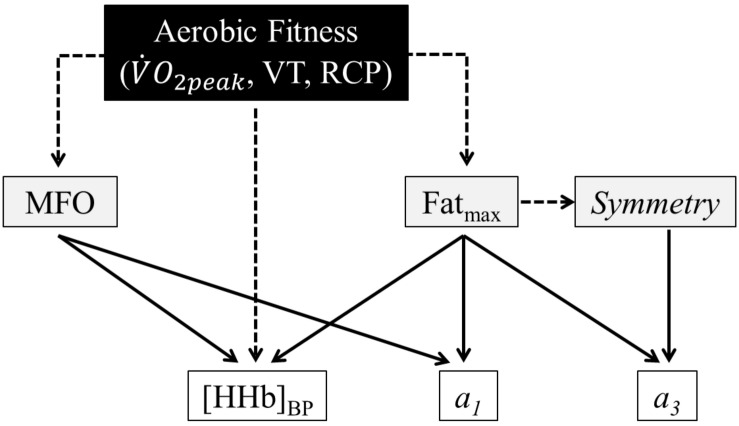
Schematic representation of the correlations among aerobic fitness [peak oxygen uptake ($\dot{V}O{}_{2\,p\,e\,a\,k}$), ventilatory threshold (VT), and respiratory compensation point (RCP)], whole-body fat oxidation kinetics [maximal fat oxidation (MFO) and exercise intensity at which MFO occurs (Fat_max_) and the *symmetry* variable], and muscle deoxygenation kinetics [the slopes of the first (*a*_1_) and second (*a*_3_) parts of the curve of the double linear model].

The correlations between whole-body fat oxidation and muscle deoxygenation parameters are listed in Table 4. MFO tended to be negatively correlated with *a*_1_ (*p* = 0.06) and was positively correlated with [HHb]_BP_ (*p* = 0.01). Fat_max_ (mL⋅kg^–1^⋅min^–1^) tended to be negatively correlated with *a*_1_ (*p* = 0.06) and was positively correlated with *a*_3_ (*p* = 0.03), [HHb]_BP_ (*p* < 0.001). When expressed in relative values (%$\dot{V}O{}_{2\,p\,e\,a\,k}$), Fat_max_ was only positively correlated with *a*_3_ (*p* = 0.01). Finally, the *symmetry* was positively correlated with *a*_3_ (*p* = 0.01). A schematic view of the correlations among aerobic fitness, whole-body fat oxidation and muscle deoxygenation kinetics is presented in Figure 3.

**TABLE 4:** Correlations between fat oxidation kinetics and muscle deoxygenation kinetics parameters.

(*n* = 22)	%Δ [HHb]		
	*a*_1_	*a*_3_	[HHb]_BP_ [mL⋅kg^–1^⋅min^–1^]
MFO [mg⋅kg^–1^⋅min^–1^]	−0.41^§^		0.66*
Fat_max_ [mL⋅kg^–1^⋅min^–1^]	−0.41^§^	0.47*	0.68*
Fat_max_ [%VO_2peak_]		0.52*	
*Symmetry*		0.58*	

Data are presented as Pearson correlation coefficients (*r*). *a*_1_ and *a*_3_, slopes of the first and second parts of the curve of the double linear model, respectively; [HHb]_BP_, breakpoint of the curve of the double linear model; Δ[HHb], variation in deoxygenated hemo- and myoglobin concentration; MFO, maximal fat oxidation; Fat_max_, intensity at which MFO occurs; $\dot{V}O{}_{2\,p\,e\,a\,k}$, peak oxygen uptake. *Significant correlation (*p* ≤ 0.05). ^§^*p* = 0.06.

## Discussion

The present study showed that the whole-body fat oxidation and muscle deoxygenation kinetics during exercise were related in individuals with different aerobic fitness levels. Cyclists exhibited (1) significant upward fat oxidation kinetics with greater MFO and absolute Fat_max_ and (2) shallower muscular deoxygenation kinetics (i.e., lower *a*_1_ values) with a right-shifted [HHb]_BP_ compared to active participants. MFO and Fat_max_ were both positively correlated with the [HHb]_BP_ and tended to be negatively correlated with *a*_1_ (Table 4 and Figure 3).

### Effect of Aerobic Fitness on Whole-Body Fat Oxidation Kinetics

Whole-body fat oxidation rates for intensities from 20 to 65% $\dot{V}O{}_{2\,p\,e\,a\,k}$ (Figure 2C) and MFO (Table 2) were greater (+39%) in cyclists than in the active group, corroborating previous studies comparing trained and very trained men (Achten and Jeukendrup, 2003; Lima-Silva et al., 2010) or trained and untrained women (Stisen et al., 2006). Furthermore, consistent with these studies, MFO was positively correlated with $\dot{V}O{}_{2\,p\,e\,a\,k}$, indicating an increased fat oxidation capacity in endurance-trained athletes. This may be due to several endurance-training adaptations, such as an increased mitochondrial volume and activity (Holloszy and Coyle, 1984; Bishop et al., 2014), an enhanced oxidative enzyme activity (Stisen et al., 2006), or a greater amount of oxidative fibers (Gollnick et al., 1972). A recent study (Shaw et al., 2020) indeed showed that MFO rate was strongly related to the proportion of type I fibers in endurance and non-endurance trained males. Fat_max_ (in mL⋅kg^–1^⋅min^–1^) was also higher in cyclists than in the active group (+29%), whereas no difference was found for Fat_max_ when expressed as relative intensity (%$\dot{V}O{}_{2\,p\,e\,a\,k}$), corroborating previous findings (Achten and Jeukendrup, 2003; Lima-Silva et al., 2010). Unlike our hypothesis, in addition to similar *symmetry* and *translation* values, the *dilatation* variable was unexpectedly greater in the active group than in the cyclists (Figure 2A and Table 2). In previous studies using similar submaximal incremental protocols and data analysis with SIN, the *dilatation* values corresponded to ∼0.1 in moderately trained men ($\dot{V}O{}_{2\,p\,e\,a\,k}$ of ∼50 mL⋅kg^–1^⋅min^–1^) (Cheneviere et al., 2011, 2012), while the mean values in an obese population (BMI > 35) ranged between -0.1 and 0.4 (Lanzi et al., 2014, 2015). According to these results, and although an averaging process for the two submaximal tests has been used to reduce the intra-individual variability of fat oxidation rates (Croci et al., 2014), the *dilatation* value seems to be more individual-dependent than training status or body composition related. Inter-individual or between-group comparisons in terms of the global shape of fat oxidation kinetics may thus be questionable and require further research. Nevertheless, the assessment of the global shape of these kinetics seems to be more sensitive and adapted to put forward differences in longitudinal study designs (Lanzi et al., 2015). However, in the present study, trained cyclists exhibited greater fat oxidation rates with significant upward fat oxidation kinetics.

### Effect of Aerobic Fitness on Muscle Deoxygenation Kinetics

In addition to greater fat oxidation, cyclists also showed a better balance between O_2_ delivery and consumption, reflected by a slower slope (*a*_1_) of %Δ[HHb] kinetics (-20%) and a [HHb]_BP_ occurring at a significantly higher absolute intensity (+42%) compared with the active group (Table 3). This was in accordance with previous studies, which found a right-shifted [HHb]_BP_ in individuals with greater $\dot{V}O{}_{2\,p\,e\,a\,k}$ (Boone et al., 2009; Murias et al., 2013; McNarry et al., 2015; Boone et al., 2016a). The strong and positive correlations found between the absolute intensity at which the [HHb]_BP_ occurred and $\dot{V}O{}_{2\,p\,e\,a\,k}$, MAP, VT, and RCP confirm the likely influence of aerobic fitness status on muscle deoxygenation kinetics. The shallower slope in cyclists indicates a lower reliance on O_2_ extraction from the blood flow by myocytes for a given $\dot{V}O{}_{2}$, reflecting a delayed reliance on glycolytic metabolism (Boone et al., 2009, 2016a). This may be due to a greater proportion of slow oxidative fibers (Gollnick et al., 1972) and/or a higher muscle capillary network (i.e., higher capillary-to-myocyte ratio) inducing a greater muscle oxidative capacity in trained athletes (Boone et al., 2016a). However, we cannot confirm these morphological differences with our measurements.

**FIGURE 4:**
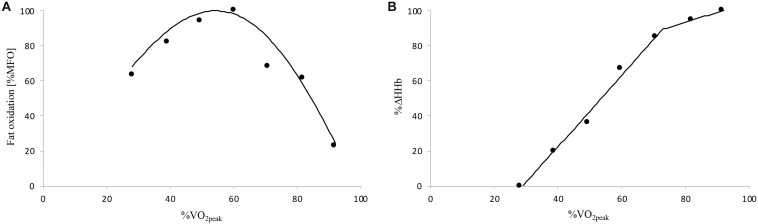
Example of the whole-body fat oxidation **(A)** and muscle deoxygenation **(B)** kinetics of a cyclist participant determined during the submaximal incremental test. Δ[HHb], variation in deoxygenated hemo- and myoglobin concentration; MFO, maximal fat oxidation; $\dot{V}O{}_{2\,p\,e\,a\,k}$, peak oxygen uptake.

It should be noted that [HHb]_BP_ determined in the present study [69% $\dot{V}O{}_{2\,p\,e\,a\,k}$ (range: 61–76) and 64% $\dot{V}O{}_{2\,p\,e\,a\,k}$ (range: 55–80) in cyclists and active individuals, respectively] occurred at lower relative exercise intensity found in previous studies. In fact, mean [HHb]_BP_ values typically ranged from ∼75 to 88% $\dot{V}O{}_{2\,p\,e\,a\,k}$ in physically active male with heterogeneous aerobic fitness level (Bellotti et al., 2013; Murias et al., 2013; Fontana et al., 2015; Boone et al., 2016a). However, all these studies determined [HHb]_BP_ during a single maximal incremental ramp test with 1-min stage duration, contrasting with the submaximal incremental test and 5-min stages used in the present study. This stage duration was needed to properly determine fat oxidation rates by ensuring $\dot{V}O{}_{2}$ and $\dot{V}\,C\,O_{2}$ steady-state values in the last minute of each step of the submaximal incremental test in both active and cyclist participants (Amaro-Gahete et al., 2019). Simultaneously, this stage duration allows us to also have a steady-state for %Δ[HHb] at each step of the submaximal incremental test and, thus, appropriately determine the relationship between whole-body fat oxidation and muscle deoxygenation kinetics during the same exercise (Figure 4). Compared to previous studies, the longer stage duration of our incremental test may have induced a lower $\dot{V}O{}_{2}$ and consequently lower %$\dot{V}O{}_{2\,p\,e\,a\,k}$for [HHb]_BP_ of our participants as previously shown for RCP (Bourdon et al., 2018; Jamnick et al., 2018). These differences in test protocol/procedure make the comparison of results difficult and may explain the present lower relative [HHb]_BP_ values compared to above studies. However, the fact that [HHb]_BP_ occurred at a higher absolute exercise intensity in individuals with greater $\dot{V}O{}_{2\,p\,e\,a\,k}$ is in accordance with previous results (Boone et al., 2009; Gravelle et al., 2012; Murias et al., 2013; McNarry et al., 2015; Boone et al., 2016a).

### Relationship Between Fat Oxidation and Muscle Deoxygenation Kinetics

In the present study, whole-body fat oxidation and muscle deoxygenation kinetics are both influenced by aerobic fitness status and seem to be dependent on the balance between O_2_ delivery and consumption, inducing a relationship between the two kinetics. This was confirmed by different correlations among the parameters of the two kinetics (Table 4 and Figure 3). In fact, MFO and Fat_max_ were positively correlated with the [HHb]_BP_ and tended to be negatively correlated with *a*_1_. This finding demonstrates that shallower muscle deoxygenation kinetics with a right-shifted [HHb]_BP_ would be associated with greater fat oxidation rates. Indeed, the adaptations induced by endurance training, such as an increased proportion of slow oxidative fibers and a developed capillarization (Hawley et al., 2014), may be responsible for a longer maintained balance between O_2_ delivery and consumption during ramp exercise (Gollnick et al., 1972; Ferreira et al., 2007; Boone et al., 2009), which provides slower muscle deoxygenation at the same absolute exercise intensity and is associated with prolonged fat oxidation during high exercise intensities. Endurance training adaptations in cyclists promote a greater peripheral muscle capacity to extract and use O_2_ to oxidize fat during incremental exercise, shifting Fat_max_ to higher absolute exercise intensities compared with active individuals. In addition, the absolute and relative values of Fat_max_, as well as the *symmetry* variable of the SIN model, were all positively correlated with the slope of the second part of the double linear model (*a*_3_). In other words, whole-body fat oxidation kinetics presenting a rightward *asymmetry*; thus, a right-shifted Fat_max_ may be associated with a steeper slope of the second part of the %Δ[HHb] kinetics. This may reflect a prolonged reliance on aerobic metabolism and thus a prolonged utilization of fat as an energetic substrate, even at high intensity when glycolytic metabolism provides more energy during exercise compared with lower intensities. In fact, our *a*_3_ values are different from zero, attesting that, at the end of the submaximal incremental test, the upper limits of the fractional O_2_ extraction were not yet reached (the test ended at 87% $\dot{V}O{}_{2\,p\,e\,a\,k}$). These correlations confirmed our hypothesis that a relationship exists between whole-body fat oxidation and muscle deoxygenation kinetics.

### Methodological Limitations

Our suggestions on the differences in fat oxidation and muscle deoxygenation between the two groups are based on possible muscular adaptations, such as differences in muscle fiber proportions. However, muscle biopsies were not taken in the present study, and we can only speculate about the likely relationship between the higher proportion of slow oxidative fibers and slower %Δ[HHb] kinetics and increased fat metabolism in cyclists compared with active individuals. Likewise, we did not measure oxidative enzyme activity, and we could thus not state whether cyclists had a greater fat oxidation because of an enhanced muscle enzyme activity (Stisen et al., 2006).

## Conclusion

Our findings confirmed the influence of aerobic fitness status on both whole-body fat oxidation (Achten and Jeukendrup, 2003; Lima-Silva et al., 2010) and muscle deoxygenation kinetics (Boone et al., 2016a), and to the best of our knowledge, this was the first study to show a relationship between the two kinetics. Our results showed that shallower %Δ[HHb] kinetics with a right-shifted [HHb]_BP_ were associated with higher fat oxidation (MFO) and Fat_max_ shifted to higher absolute exercise intensities. This corroborates that, compared to active individuals, endurance-trained cyclists may maintain aerobic ATP production until higher exercise intensities and may have a delayed reliance on glycolytic metabolism because of a longer maintained balance between O_2_ delivery and consumption, supporting higher fat oxidation rates.

## Data Availability Statement

The datasets generated for this study are available on request to the corresponding author.

## Ethics Statement

The studies involving human participants were reviewed and approved by Commission cantonale d’Éthique de la Recherche sur l’être humain Vaud (CER-VD)—Swissethics. The patients/participants provided their written informed consent to participate in this study.

## Author Contributions

AZ, XC, and DM conceived and designed the research study. AZ, SL, CT, NB, XC, and DM performed the experiments. AZ, SL, LV, CT, XC, and DM analyzed the data. AZ, BG, BK, XC, and DM interpreted the results of the experiments. AZ, XC, and DM prepared figures and drafted the manuscript. All authors approved the final version of the manuscript.

## Conflict of Interest

The authors declare that the research was conducted in the absence of any commercial or financial relationships that could be construed as a potential conflict of interest.
